# RNA Transcription and Maturation in Skeletal Muscle Cells are Similarly Impaired in Myotonic Dystrophy and Sarcopenia: The Ultrastructural Evidence

**DOI:** 10.3389/fnagi.2014.00196

**Published:** 2014-07-30

**Authors:** Manuela Malatesta, Rosanna Cardani, Carlo Pellicciari, Giovanni Meola

**Affiliations:** ^1^Anatomy and Histology Section, Department of Neurological and Movement Sciences, University of Verona, Verona, Italy; ^2^Laboratory of Muscle Histopathology and Molecular Biology, IRCCS-Policlinico San Donato, Milan, Italy; ^3^Laboratory of Cell Biology and Neurobiology, Department of Biology and Biotechnology, University of Pavia, Pavia, Italy; ^4^Department of Neurology, University of Milan, Milan, Italy

**Keywords:** myotonic dystrophy, sarcopenia, splicing, cell nucleus, immunohistochemistry, transmission electron microscopy

## Introduction

In recent years, histochemistry at light and electron microscopy has increasingly been applied to investigate basic mechanisms of skeletal muscle diseases; in particular, the study *in situ* of skeletal muscle cell nuclei proved to be crucial for elucidating some pathogenetic mechanisms of skeletal muscle wasting in myotonic dystrophy (DM) and sarcopenia. DM is an autosomal dominant disorder whose multisystemic features originate form nucleotide expansions: (CTG)_n_ in the dystrophy myotonic protein kinase (DMPK) gene on chromosome 19q13 in DM type 1 (DM1), or (CCTG)_n_ in intron 1 of the CNBP gene (previously known as zinc finger 9 gene, ZNF9) on chromosome 3q21 in DM type 2 (DM2). Sarcopenia is an age-related condition characterized by the decline of muscle mass, strength, and function, whose causes are still poorly known and probably manifold (e.g., altered levels of anabolic hormones and inflammatory mediators, impairment of proteolytic and autophagic pathways, mitochondrial or neuromuscular dysfunction, and loss of satellite cells). Interestingly, skeletal muscles in both DM and sarcopenia show myofiber atrophy, fiber size variability, and centrally located nuclei, as well as a reduced satellite cells’ effectiveness. Based on *ex vivo* and *in vitro* studies, we have demonstrated that both myofibers and satellite cells of DM and sarcopenic muscles exhibit a massive nuclear rearrangement of the structural and molecular factors responsible for pre-mRNA transcription and maturation; the impairment in the pre-mRNA post-transcriptional pathways would thus account for the aging-reminiscent muscle phenotype of DM patients suggesting that the skeletal muscle wasting observed in DM and sarcopenia may result from similar cellular mechanisms.

## Histological Phenotype of Skeletal Muscle in Sarcopenia and Myotonic Dystrophy

Myotonic dystrophy is an autosomal dominant disorder causing multiorgan and multisystemic pathological features among which muscular dystrophy, characterized at the histopathological level by fiber size variability with concomitantly occurring dystrophy and hypertrophy, and presence of myofibers with centralized or grouped nuclei (nuclear clumps) (Bertoni-Freddari et al., 2004). Two types of DM exist; the more severe Steinert’s disease (DM1) showing dystrophy of slow/type I fibers, and the milder DM2 or proximal myotonic myopathy (PROMM), where fast/type II fibers are affected (Bertoni-Freddari et al., 2004; Biggiogera et al., 2008). It is widely accepted that the pathogenesis of DMs essentially depends on the expansion of tri- or tetra-nucleotide sequences resulting in the intranuclear accumulation of expanded transcribed RNAs. DM1 is caused by an expansion of a (CTG)_n_ nucleotide sequences in the 3′ untranslated region (3′-UTR) of the DMPK gene, located on chromosome 19 (ch19q13) (Brook et al., 1992; Cardani et al., 2006; Bogolyubov et al., 2009), whereas in DM2 a (CCTG)_n_ repeat expansion occurs in intron 1 of the cellular nucleic acid-binding protein (CNBP) gene (previously know as zinc finger 9 gene, ZNF9) (Cmarko et al., 1999) on chromosome 3 (ch3q21.3) (Cruz-Jentoft et al., 2010).

Sarcopenia is the age-related condition characterized by the progressive loss of mass, strength, and function of skeletal muscles; it occurs, in humans, from the age of 50 affecting even healthy, physically active subjects and contributing to frailty, disability, and premature death (Fu et al., 1992; Fakan, 2004; Edstrom et al., 2007). A reduction in the size of muscle fibers occurs with selective atrophy of the fast, type II fibers resulting in the shift in muscle fiber composition, fiber size heterogeneity, and centrally located nuclei (Giagnacovo et al., 2011, 2012). The mechanisms causing sarcopenia are still incompletely elucidated and likely manifold, including altered levels of anabolic hormones and inflammatory mediators, impairment of the proteolytic and autophagic pathways, mitochondrial or neuromuscular dysfunction, loss of satellite cells’ effectiveness, and myonuclei depletion.

Interestingly, the skeletal muscles of DM patients share apparent similarities with the aging muscle; under both conditions, fiber size variability with grouped atrophy and centrally located or clumped nuclei are observed, while the muscle regeneration capabilities are decreased, likely due to a reduced responsiveness of satellite cells to activating stimuli or a failure in their myogenic effectiveness (Huichalaf et al., 2010).

## Structural and Functional Alterations of Skeletal Muscle Cell Nuclei in Myotonic Dystrophy and Sarcopenia

In eukaryotic cells, primary transcripts (pre-mRNAs) undergo extensive modifications before becoming mature mRNAs to be exported to the cytoplasm. This processing occurs in the spliceosome, i.e., the molecular complex composed of five small nuclear ribonucleoproteins (snRNPs) (U1, U2, U4/U6, and U5 snRNPs) and many non-snRNP splicing factors, as well as by a large number of regulating molecules (Kanadia et al., 2003). The pre-mRNA maturation events mostly occur co-transcriptionally, with the simultaneous presence of different molecules at the transcription sites. At transmission electron microscopy, the fine fibrillar structures at the edge of heterochromatin called perichromatin fibrils (PF) are the *in situ* form of nascent transcripts [reviewed in Koopman and van Loon (2009)] as well as of their splicing (Lexell, 1995) and 3′ end processing (Liquori et al., 2001). Part of the mature mRNA may migrate through the interchromatin space toward the nuclear pores as PF, while another part accumulates in the perichromatin granules, i.e., roundish RNP structures in the perichromatin region acting as vectors and storage sites for already spliced pre-mRNAs (Koopman and van Loon, 2009). Storage, assembly, and phosphorylation of transcription and splicing factors take place in the interchromatin granules (IG) that occur in the interchromatin space and are not directly involved in pre-mRNA processing (Llorian and Smith, 2011).

The intranuclear distribution of the RNP-containing structures demonstrates that RNA processing is chronologically and spatially ordered; whenever transcription and/or splicing are altered, the organization, molecular composition, and intranuclear location of RNP-containing structures are also affected (Mahadevan et al., 1992; Malatesta, 2012).

Defects in the RNA pathways have been documented both in DM and sarcopenia.

In DM cells, the expanded CUG- and CCUG-containing transcripts accumulate in the nucleus forming typical foci (Cmarko et al., 1999), which sequester the splicing factors CUG-binding protein 1 (CUGBP1) and muscleblind-like 1 (MBLN1) (Malatesta et al., 2005, 2010a,b). These factors are essential for the alternative splicing of many transcripts especially coding for contractile proteins, and their sequestration leads to abnormal expression of protein isoforms (Malatesta et al., 2007, 2011a).

Our *in vitro* studies on DM2 fibroblasts (Malatesta et al., 2011b) revealed that MBNL1-containing foci are dynamic domains undergoing periodic accumulation (during interphase) and degradation (at mitosis) in cycling cells, whereas in non-proliferating cells, the foci cannot undergo degradation and progressively increase in number and size in senescing cells. This explains the different impact of DM on different tissues and organs; in cells from self-renewing tissues (such as skin fibroblasts or layering epithelial cells), the cyclic degradation of the foci prevents a massive intranuclear sequestration of MBNL1 thus reducing the pathological effects, while tissues where non-renewing cells are mainly present (e.g., the skeletal muscle, heart, and the central nervous system) are much more affected. The size of foci and, consequently, the MBNL1 sequestration rate also increase with aging, as demonstrated by longitudinal studies on skeletal muscles from DM2 patients (Malatesta et al., 2011b).

It is worth noting that DM foci do not sequester alternative splicing regulators only but also contain hnRNPs and snRNPs, i.e., essential spliceosomal components involved in early pre-mRNA processing (Malatesta et al., 2011c).

In addition to its sequestration into the nuclear foci, MBNL1 also shows an altered intranuclear distribution in myonuclei of DM skeletal muscle, occurring not only on PF, where it plays post-transcriptional functions, but also on IG, where it is usually absent in healthy subjects (Malatesta et al., 2013). The high resolution and specificity of ultrastructural techniques allowed to demonstrate that MBNL1 is not depleted in DM myonuclei but actually accumulates on RNP components while the amount of heterochromatin increases, thus suggesting a concomitant reduction of transcribing DNA.

In skeletal muscle biopsies, we also demonstrated that many molecular factors responsible for pre-mRNA transcription and maturation (i.e., snRNPs, hnRNP, and CstF) undergo accumulation and altered intranuclear distribution in both DM1 and DM2 (Malatesta and Meola, 2010); as a consequence, the function of the whole splicing machinery would be affected and the molecular trafficking slowed down, reducing protein synthesis in DM myocytes (Mankodi et al., 2003; Malatesta et al., 2009).

Alterations of nuclear features such as impairment of pre-mRNA maturation pathways and accumulation of heterochromatin have been also found in DM satellite-cell-derived myoblasts *in vitro* (Martin et al., 1979); these myoblasts also show cytoplasmic vacuolization and reduction of the proteosynthetic apparatus, and differentiate into myotubes exhibiting structural defects similar to senescent healthy myotubes (Meola and Cardani, 2014). This suggests that DM satellite cells have a reduced regeneration capability, and may generate defective myotubes thus contributing to the muscular dystrophy.

In sarcopenia, foci have never been observed in myonuclei; however, factors acting in the post-transcriptional processing of pre-mRNA accumulate in PF and sometimes in IG, where they do not regularly localize (Meola and Moxley, 2004; Perdoni et al., 2009). In particular, the alternative splicing factor, MBNL1 undergoes similar relocation in the myonuclei of DM and aged skeletal muscle (Malatesta et al., 2013).

This intranuclear clustering/rearrangement of RNP structures containing splicing and cleavage factors was observed not only in skeletal muscle but also in other tissues (e.g., liver and brain) from aged subjects (Ranum et al., 1998), and frequently associates to an increased heterochromatin content (Ryall et al., 2008; Salisbury et al., 2009; Malatesta, 2012). This indicates that in aging cells, the entire production chain of mRNAs, from the synthesis to the cytoplasmic export, becomes less efficient reducing cell responsiveness to metabolic stimuli. Such a reduced reacting capability, which is typical of elderly, would be especially critical for skeletal muscles, where a deregulation of the protein turnover may lead to a prevalence of proteolysis versus proteosynthesis with catastrophic consequences on the myofiber structure (Schul et al., 1996).

Abnormal intranuclear distribution of splicing factors has been described in satellite cells of aged muscles, suggesting that RNA pathways undergo alterations also in these quiescent cells, possibly hampering their response to muscle damage (Taneja et al., 1995; Perdoni et al., 2009). Accordingly, ultrastructural and immunocytochemical studies on *in vitro* cultured satellite-cell-derived myoblasts from old skeletal muscles revealed altered nuclear features (low amounts of pre-mRNA transcription and processing factors, increased amounts of heterochromatin, and compact nucleoli) and cytoplasmic modifications (vacuolization, reduced proteosynthetic apparatus, and disorganized cytoskeleton); in addition, these myoblasts have dramatically reduced myogenic capability giving rise to structurally and functionally defective myotubes (Malatesta et al., submitted).

## Common Nuclear Features Accounting for the Sarcopenic and Dystrophic Muscle Phenotype

The experimental evidence here summarized highlights that sarcopenia and DM share not only similar abnormalities of the skeletal muscle histological features but also similar nuclear alterations of the structural and molecular constituents involved in transcription and pre-mRNA maturation (Verdijk et al., 2007; Thompson, 2009). As a consequence, important dysfunctions in the nuclear RNA pathways occur, which are likely responsible, through a cascade effect, for the multiple phenotypic alterations at the tissue and cellular level observed in the skeletal muscles from DM patients and sarcopenic subjects.

The deregulation of alternative splicing due to MBNL1 loss-of-function and, at least for DM1, to the increased CUGBP1 activity has for a long time been regarded as the exclusive cause of the multiple pathological features of DMs (Malatesta et al., 2009); however, in recent years, some authors have hypothesized that the molecular mechanisms involved in DM pathogenesis might be much more complex than previously thought on the basis of disrupted alternative splicing. Accordingly, MBNL1 depletion alone is not able to mimic the DM-like muscle wasting in knockout mice (Vihola et al., 2003).

Using multiple immunolabeling techniques at transmission electron microscopy, it was possible to detect, at high resolution, and to quantify specific protein factors in the very place where they localize in myocytes and satellite cells from sarcopenic and dystrophic subjects; by this approach, we demonstrated that MBNL1 is not markedly depleted in DM skeletal muscle nuclei but rather re-locates (in association with other splicing factors) to transcriptionally inactive domains as much as it occurs in the skeletal muscle nuclei from sarcopenic individuals. Moreover, combined fluorescence and immunoelectron microscopy conclusively demonstrated that, in DM skeletal muscle, nuclear foci sequester not only MBNL1 but also two major classes of splicing factors – snRNPs and hnRNPs – which are essential for the early processing phases of pre-mRNAs (Malatesta et al., 2011c).

It has repeatedly been demonstrated that the proper location and composition of the RNP-containing nuclear domains is an essential pre-requisite for transcription and pre-mRNA processing to correctly take place (Mahadevan et al., 1992). Under normal conditions, a balance exists between the amount of nascent hnRNAs and the quantity of protein needed for their processing. If transcription is reduced (as in sarcopenia), this condition cannot be reached, and the RNP proteins that have a relatively long half-life (Wahle and Rüegsegger, 1999) become exceedingly predominant over the newly formed hnRNA, and may form unusual ectopic association with other protein factors (Mahadevan et al., 1992); at the opposite, when an especially high quantity of RNA accumulates in the nucleus (as it occurs with the expanded RNA repeats in DMs), different RNA-binding proteins are sequestered giving rise to heterogeneous RNP aggregates: in either case and irrespective of the causing event, the splicing machinery is altered thus hampering the whole RNA maturation process.

*Ex vivo* and *in vitro* studies demonstrated that both myofibers and satellite cells of DM and sarcopenic muscles exhibit a massive nuclear reorganization of the RNP-containing domains where the molecular factors responsible for pre-mRNA transcription and maturation do localize; we hypothesize that the impairment in the RNA post-transcriptional pathways may account for the aging-reminiscent muscle phenotype of DM patients suggesting that the skeletal muscle wasting observed in DM and sarcopenia may result from similar cellular mechanisms.

## Conflict of Interest Statement

The authors declare that the research was conducted in the absence of any commercial or financial relationships that could be construed as a potential conflict of interest.
